# What is communicative competence and how can it be acquired?

**DOI:** 10.3205/zma001445

**Published:** 2021-03-15

**Authors:** Claudia Kiessling, Götz Fabry

**Affiliations:** 1 Universität Witten/Herdecke, Fakultät für Gesundheit, Lehrstuhl für die Ausbildung personaler und interpersonaler Kompetenzen im Gesundheitswesen, Witten, Germany; 2 Albert-Ludwigs-Universität, Medizinische Fakultät, Medizinische Psychologie & Medizinische Soziologie, Freiburg, Germany

**Keywords:** communicative competence, communication skills, learning theory, acquisition of communication skills, transfer

## Abstract

The commentary deals with the question of what constitutes communicative competence (or communication skills) and to what extent findings regarding motor and social skills are transferable to the domain of communication. After a proposal for a definition, the commentary considers how learners acquire communicative competence and what needs to be considered from the trainers’ perspective in order to support learners in their competence development.

The commentary does not claim to present all definitions of the concept of competence or communicative competence in a comprehensive way. Nor does it aim to present the current state of research. Our aim is to provide teachers and interested individuals in medical education with a pragmatic guide to how communicative competence can be taught and learned based on a skills model.

## Zusammenfassung

Der Kommentar setzt sich mit der Frage auseinander, was kommunikative Kompetenz konstituiert und inwieweit Erkenntnisse bezüglich motorischer und sozialer Fertigkeiten auf die Domäne Kommunikation übertragbar sind. Nach einem Vorschlag für eine Definition erfolgen Überlegungen, wie Lernende kommunikative Kompetenz erwerben und was dabei aus Sicht der Dozierenden zu beachten ist, um Lernende bei ihrem Kompetenzaufbau zu unterstützen.

Der Kommentar erhebt nicht den Anspruch, sämtliche Definitionen des Kompetenzbegriffs oder kommunikativer Kompetenzen umfassend darzustellen. Es geht auch nicht um die Darstellung des aktuellen Forschungstands. Unser Ziel ist eine pragmatische Handreichung für Lehrende und Anwender*innen in der medizinischen Ausbildung, wie – basierend auf einem Fertigkeiten-Modell – kommunikative Kompetenz vermittelt und gelernt werden können.

## 1. Introduction

Communicative competence (or communication skills) is one of the central prerequisites for successful medical practice, of this there is probably no serious doubt [1]. However, there is less agreement whether and, if so, how communicative competence can be acquired [2], [3]. The answer to this question also depends on what we mean by communicative competence. In the literature, there is now a large number of explanatory models from various disciplines (e.g. medicine, psychology, sociology, linguistics, university didactics, communication sciences) that attempt to answer this question. A complete presentation of these explanatory models would go beyond the scope of this commentary. 

We believe that it makes sense, particularly with regard to educational issues, to understand communicative competence as the situation-specific use of communicative skills [4]. Thus, the scientific findings on the teaching, acquisition and testing of skills are also valid for the field of clinical communication. 

## 2. What is communicative competence?

When we deal with the question of whether and how we should teach communicative competence, we must first define what communicative competence actually is. Unfortunately, as in the discussion about the concept of competence in general, there is no uniform definition. In the scientific literature, no systematic or clear distinction is made between “Communication Competence” and “Communication Skills”. For example, the standard work by Silverman, Kurtz and Draper is entitled “Skills for Communicating with Patients”, although it is probably the aim of the book to promote the acquisition of communicative competence even though the term “competence” does not appear in the book [5]. Thus, for pragmatic reasons it seems obvious and legitimate to use the concept of skills when we search for a definition of communicative competence and to ask the question of whether and, if so, what similarities exist between motor skills, social skills and communication skills.

According to Owen Hargie, who has developed a comprehensive concept of communication skills, a motor skill is an organised, coordinated, goal-oriented activity that involves a sequence of sensory, cognitive and motor mechanisms [6]. A skill can be learned, i.e. understanding and behaviour are built up step by step through repeated experience. A skill is also serial, i.e. there is an order and coordination of different processes and activities in a sequence.

However, perhaps a communication skill is less a motor skill than an interactional or social skill. Hargie defines a social skill as a process in which an individual applies a set of purposeful, interconnected, situational appropriate social behaviours that are learned and controlled [6]. Social skills include verbal and non-verbal behaviour, involve appropriate prompts and responses that require timing and control of specific behaviour, and are influenced by contextual factors. Therefore, the process of couple dancing is an apt metaphor for social skills [7]: Each dancing partner brings with him/her own motives, e.g. to impress the other or to build a relationship. Couples complement each other in fluid, coordinated patterns. This requires a coordinated interlocking of the learned repertoire of both partners. Certain “moves” are expected or anticipated. If one cannot dance or tries a different dance step, it becomes difficult. 

So where are the differences between motor and social skills? Social interaction is related to other people, i.e. one's own goals and the goals of others are included, and rules and routines are less strictly defined. As far as social skills are concerned, emotions and personal involvement play a greater role (self-esteem, acceptance, etc.). Perception in the process plays a greater role, as it is not only about the perception of the skill itself, but also about one's own reactions and the reactions of others. And personal factors also play a greater role (age, gender, origin etc.). A first hypothesis at this point would be that communicative competence is more similar to social skills than to motor skills [6].

Let us now look at the competence definition. By now a large number of definitions of the term competence do exist. In the didactic context of higher education, reference is often made to the Weinert’s definition, who describes competencies as ”the cognitive abilities and skills available to individuals or learnable by them to solve certain problems, as well as the associated motivational, volitional and social readiness and ability to use problem-solving in variable situations successfully and responsibly” [8]. Competences are therefore highly specific and can best be understood from the requirements side: We experience and evaluate a person as competent when he or she is able to solve certain tasks or meet certain requirements. Skills, on the other hand, can be understood as specific behavioral or action dispositions that must be organized and used in a situationally appropriate manner when solving a non-trivial task.

## 3. Proposal for a definition of communicative competence

If one now tries to bring together the ideas on social skills according to Hargie with the concept of competence according to Weinert, one could define communicative competence as follows: 

Communicative competence is the ability to achieve communicative goals in a socially appropriate manner. It is organized and goal-oriented, i.e. it includes the ability to select and apply skills that are appropriate and effective in the respective context. It includes verbal and non-verbal behaviour. The application of communicative competence is serial, i.e. different processes and activities are coordinated in a typical sequence and require appropriate timing and control of specific behaviour. It is influenced by the behaviour of the other person and by the context and requires the preparedness and willingness to communicate with the other person for the benefit of all. Communicative competence can be acquired, i.e. the necessary skills are built up step by step through repeated, reflected practice and experience [9], [10] quoted from [6].

## 4. How can learners acquire communicative competence?

When acquiring motor or cognitive skills, the prior experience of the learner is of central importance, because the design of the learning environment must be based on it [11], [12]. A learner with little experience must first get an overview and then build up a knowledge base (e.g. by reading, discussions etc.), while application initially plays a subordinate role. More experienced learners at an intermediate level benefit from worked examples and can focus more on specific problem solving [13]. In this way, experience-based knowledge and heuristics are built up and misconceptions can be corrected. High-level learners with a lot of prior experience can improve the accuracy and speed of their skills through repeated practice (Deliberate Practice) and transfer them to other contexts [14]. These considerations are also plausible against the background of findings on the use of cognitive resources (cognitive load) [15]: Since individual cognitive resources are basically limited, learning tasks should be designed in such a way that they are adapted to the level of expertise of the learners [16]. This can be illustrated well by the example of simulations, which are often used to train communication skills. In order to take the patient’s history in a patient-centred manner, a student must know what to ask (the content of the history), listen attentively to the patient and control his or her conversational behaviour (e.g. ask open questions). At the same time however, he or she must pay attention to the patient's behaviour in order to notice emotional cues, for example, to which he or she can then react empathically (which in turn requires him or her to know how to do this). It is easy to imagine that students with little prior experience are quickly overwhelmed in such a situation because they have to consciously process many tasks, while experienced students at least partially have automated routines and can thus free up more cognitive resources, e.g. to deal with demanding content in a medical encounter (see [17]).

Other important findings on the acquisition of skills relate to the transfer. First of all, it has been shown time and again that learners find it very difficult to transfer skills they have acquired in one context to another content domain. In order to better understand what this transfer is about, a distinction can be made between forward transfer (anticipating clinical practice while learning) and backward transfer (remembering learning when working clinically). In the case of backward transfer, it must be recognised that the current situation has similarities with the situation that has already been successfully mastered in the past [18]. Therefore, it is important to promote forward transfer in studies by considering as many and different application contexts as possible already during learning, so that later backward recognition is easier. In principle, a close transfer that involves similar content (e.g., reacting empathetically to emotional reactions of children and adults) is easier to achieve than a distant transfer that involves different content areas: information-giving in medical conversations for instance, may have different requirements needing different skills in different contexts (e.g., conversations regarding curative vs. palliative treatment).

With regard to the above-mentioned matching of learning environment and level of expertise, a further distinction is useful: We speak of inward transfer when learning is facilitated by what has already been learned. This is the case, for example, when basic anatomical or biochemical knowledge can be used to learn basic clinical concepts (“Preparation for future Learning”) [19]. On the other hand, an outwardly directed transfer occurs when learning directly results in better problem-solving in clinical practice [20]. This distinction is important because, especially with little prior knowledge or experience, an outward transfer cannot be expected directly, but only indirectly when the corresponding knowledge structures closer to the problem have been established. Therefore, learning contents should not be judged hastily only by their usefulness for outward transfer.

By now, there is also some evidence on how transfer-promoting learning should look like [21]. First of all, a skill that is better mastered can be transferred more easily. For example, a student who has no difficulty whatsoever in responding empathically to emotional cues from his patients when taking a history will also be able to do so relatively quickly in medical conversations focused on information-giving even if the content or other requirements are very demanding. Of course, this presupposes that sufficient learning time was available to learn the respective skill accordingly, which is often not the case in the practice of medical studies. The context-bound nature of skills can be counteracted by using contrasting examples from different content areas from the outset to illustrate deeper principles.

## 5. Conclusion

With the certainly incomplete considerations outlined here, we want to make clear that the understanding of communicative competence can benefit from understanding it as the situation-specific use of specific skills. We might then transfer what we already know about the acquisition of motor and other skills to the domain of communication, and this knowledge can help us to systematically train communicative competence. Of course, we need more scientific evidence for this claim, for example to check whether the transfer of knowledge postulated here, e.g. from the domain of clinical reasoning or motor skills, is actually permissible. We also need more applied research on various teaching formats, for example with regard to the longitudinal development and acquisition of communication skills in both initial and continuing education and training. In the age of experiential learning, perhaps the question of what knowledge base is conducive or even necessary for the acquisition of communicative competencies has also been neglected. Even if this is only a personal statement, it is worth considering whether the learning environments that we are currently creating provide optimal support for learners to develop communicative competence in a sustainable way.

## Notes

The commentary is based on a presentation given by the authors on 10.05.2019 at the KUSK Workshop 2019 in Grünberg (Germany). 

## Competing interests

The authors declare that they have no competing interests. 
